# Healthcare provider interventions to support medication adherence: state-of-the-science overview

**DOI:** 10.3389/fphar.2025.1567967

**Published:** 2025-07-22

**Authors:** Jacob Crawshaw, Nicola McCleary

**Affiliations:** 1 Methodological and Implementation Research Program, Ottawa Hospital Research Institute, Ottawa, ON, Canada; 2 Child Health Evaluative Sciences Program, The Hospital for Sick Children, Toronto, ON, Canada; 3 Institute of Health Policy, Management and Evaluation, University of Toronto, Toronto, ON, Canada

**Keywords:** medication adherence, behaviour change, healthcare providers, implementation science, health systems, state-of-the-science

## Abstract

Medication adherence remains a global health issue and healthcare providers (HCPs) play an important role in supporting patients to adhere to treatment. This article provides a state-of-the-science overview of the evidence for: i) the effectiveness of HCP-delivered interventions on medication adherence outcomes; and ii) the types of implementation approaches targeting evidence-to-practice gaps among HCPs supporting medication adherence. Hundreds of randomized controlled trials and dozens of systematic reviews on the effectiveness of HCP-delivered interventions have been conducted to date. HCP-delivered interventions typically produce small-to-medium effect sizes on adherence outcomes, however, there is considerable heterogeneity in effects and few interventions that show promise are implemented into routine practice. Some key features of potentially effective HCP-delivered interventions include: moving beyond education-only, using multiple behaviour change strategies, tailoring interventions to different determinants of non-adherence, incorporating pharmacists and nurses to deliver interventions, providing ongoing support to patients, and addressing health system-level barriers and inequities. To improve the uptake of evidence into adherence-related clinical practice, it is likely that health systems must adapt to enable HCPs to better support adherence over time and in a patient-centered way. Such approaches include, improving routine screening of adherence issues, making adherence-related clinical guidelines more actionable, using routinely collected data to identify patients with adherence challenges, enhancing HCP incentivization models, and establishing quality indicators for adherence monitoring and support. Concepts and evidence from implementation science should be leveraged to support these types of system-level approaches to address evidence-to-practice gaps. In conclusion, despite an extensive evidence base for the effectiveness of HCP-delivered interventions - and a growing body of evidence for approaches targeting practice change among HCPs - we have identified several areas that could help advance the field. These include optimizing the content and delivery of adherence interventions, understanding how to implement effective strategies, and reaffirming the need for health system-level solutions.

## 1 Introduction

Medication adherence is a global health problem which has been extensively researched over the past 60 years. Taking medication as prescribed is crucial for the full benefits of the therapy to be realized, yet many patients face challenges in this regard which can lead to poorer clinical outcomes, increased healthcare utilization, and additional cost to health systems (Khan and Socha-Dietrich, 2018; Sabaté, 2003). Medication non-adherence is considered a major problem across all chronic conditions with myriad factors associated with poor adherence identified from the literature (e.g., patient-, disease-, therapy-, socioeconomic-, and healthcare system-related factors (Sabaté, 2003; Kardas et al., 2024)). Not only does this reflect the complexity of medication-taking as a behaviour (i.e., there are many potential barriers to taking medication as prescribed), it also means that it can be difficult to identify the key issues among individual patients having difficulties with their regimen (Kardas et al., 2013).

Medication adherence is defined as the extent to which a patient takes a medication in line with the treatment regimen agreed upon with their healthcare provider (HCPs) (Sabaté, 2003). Medication-taking behaviour can be difficult to measure in routine practice and often relies on self-report from patients which is associated with potential social desirability and recall bias that may underestimate the extent of the problem. Moreover, HCPs have been shown to underestimate rates of non-adherence among their patients (MacIntyre et al., 2005; Miller et al., 2002) meaning that patients who may need support can often go undetected. Researchers have posited three stages of medication adherence: initiation (e.g., starting a medication), implementation (e.g., fitting medication-taking into one’s routine), and persistence (e.g., maintaining medication-taking over time) (Vrijens et al., 2012). Barriers to medication-taking may look very different depending on the stage of adherence. For example, understanding how to take a medication correctly is particularly important during the initiation phase, understanding where a medication best fits into one’s daily routine is important during the implementation phase, and connecting with a HCP if there are concerns about side effects may be a necessary action during the maintenance phase.

Several behaviour change theories, models, and frameworks have been applied to better our understanding of medication-taking behaviour (Conn et al., 2016a). One such prominent theory of medication adherence is the Perceptions and Practicalities Approach (PAPA) developed by Horne and colleagues (Horne et al., 2019). The PAPA posits that individuals taking medication can experience both perceptual (e.g., patients’ beliefs and preferences about their medication regimen–intentional non-adherence) and practical barriers (e.g., patients’ capacity and resources to follow their medication regimen–unintentional non-adherence) and that any support provided to patients should match the types of barriers they are experiencing (National Institute for Health and Care Excellence, 2009). For example, if a patient is weighing up whether a medication is going to help their condition (necessity beliefs) versus the risk of problematic side effects (concerns about adverse effects), this would be considered a perceptual barrier. Where a patient is having difficulty following a medication regimen due to an inconvenient dosing schedule, this would constitute a practical barrier. The type of patient-centered supports offered by HCPs are likely to differ markedly depending on the type of barrier identified with some designed to make adherence easier and more convenient and others to enhance motivation by addressing the perceptions that influence motivation (National Institute for Health and Care Excellence, 2009).

HCPs play a crucial role in supporting patients to take their medications as prescribed. HCPs are the gatekeepers for prescribed medications and their interactions are central for setting patients up for success with their treatment regimens. Different HCPs are involved in the prescribing process and supporting medication-taking over time. Physicians, nurses, and pharmacists can all play key roles to support adherence across a patients’ journey, however, there are inconsistencies in how such roles are fully realized in routine practice and multiple barriers in medication adherence management continue to be surfaced in the literature (Hafez et al., 2024). In particular, issues can arise when medication adherence is not seen as a shared goal and responsibility between HCP and patient which can undermine efforts to help patients take medication correctly over time (Bosworth et al., 2011).

HCPs can be considered as either intervention deliverers (e.g., a pharmacist providing a standardized counselling session to a patient about the importance of adherence) or intervention recipients (e.g., conducting an audit of practice among pharmacists and providing feedback (i.e., audit and feedback) to identify opportunities to improve practice), which is a subtle but important distinction. This perspective can also be extended to consider the ‘dual’ role of HCPs within the same intervention study. For example, in studies where HCPs are intervention deliverers, they should also be considered as intervention recipients and work should be done to understand their barriers to change and what can then be done to support implementation. We believe it is crucial to identify the supports that HCPs themselves need to change their clinical behaviour to increase the likelihood that patients receive evidence-based care to support medication-taking. To achieve this, we can draw upon concepts and evidence from implementation science which is a discipline focused on understanding why evidence-to-practice gaps occur in healthcare and how such gaps can be addressed in the real world (Grimshaw et al., 2012).

## 2 Aims

The aim of this state-of-the-science overview is two-fold. First, we will summarize evidence from a suite of systematic reviews looking at the effectiveness of HCP-delivered interventions on medication adherence outcomes. Second, we will take concepts and evidence from implementation science and summarize evidence on approaches targeting evidence-to-practice gaps among HCPs supporting medication adherence. In this overview, we focus mainly on data from randomized controlled trials, systematic reviews, and meta-analyses rather than individual studies or other types of intervention study designs.

## 3 Impact of HCP-delivered interventions on medication adherence outcomes

There have been dozens of systematic reviews (and systematic review of reviews) looking at the effectiveness of HCP-delivered interventions on medication adherence outcomes (Wilhelmsen and Eriksson, 2019; Anderson et al., 2020; Ryan et al., 2014). As an exploratory exercise, we conducted a search of the Cochrane Library - considered the gold standard for evidence synthesis studies - to identify systematic reviews of interventions targeting medication adherence which likely reported features of HCP-delivered interventions (note, given this was an informal scan of a singular evidence repository, we do not report key information such as inclusion/exclusion criteria and PRISMA flowchart as per systematic review guidance). A total of 68 systematic reviews from the Cochrane Library had the term “medication adherence” listed in the title/abstract or as a keyword. Among this suite of systematic reviews, we screened for findings related to features of HCP-delivered interventions on medication adherence outcomes. We found 10 studies which reported key features of interventions which are summarized in Table 1. Among such studies, a range of clinical and health system outcomes were found including mortality, morbidity, healthcare utilization, healthcare costs, patient satisfaction, and quality of life (Conn et al., 2016b).

**TABLE 1:** Select studies from the Cochrane Library reporting features of HCP-delivered interventions to support medication adherence.

Author	Population	Intervention	Comparison	Outcome	Number of studies	HCP group	Key features of HCP-delivered interventions	Effect sizes	Certainty of evidence (based on GRADE criteria)
Nieuwlaat et al. (2014)	Various	Interventions of any sort intended to affect adherence with prescribed, self-administered medications	Group not receiving the intervention	Medication adherence Clinical outcomes	182 RCTs	Allied health providers	“The RCTs at lowest risk of bias generally involved complex interventions with multiple components, trying to overcome barriers to adherence by means of tailored ongoing support from allied health professionals such as pharmacists, who often delivered intense education, counseling (including motivational interviewing or cognitive behavioural therapy by professionals) or daily treatment support (or both), and sometimes additional support from family or peers”	Not conducted due to high heterogeneity	Not reported
Cross et al. (2020)	Older adults	Interventions to improve medication-taking ability or medication adherence	Usual care or receiving a different intervention	Medication adherence Medication-taking ability	50 studies	Pharmacists (31 studies) Nurses (17 studies) Physicians (15 studies)	“When considered separately by subgroups based on health professional delivering the intervention, there was no difference in adherence between those interventions delivered by pharmacists, nurses, or two or more health professionals when measured either as a dichotomous outcome”	Dichotomous outcome (risk ratio 1.21 versus 1.19 versus 1.38; test for subgroup differences p = 0.83; I^2^ = 0%) Continuous outcome (standardized mean difference = 1.38 versus −0.13 versus 0.42; test for subgroup differences p = 0.08; I^2^ = 61.4%)	Low
Al-Aqeel et al. (2020)	Epilepsy	Effectiveness of interventions aimed at improving adherence to antiepileptic medication in adults and children with epilepsy	Usual care or no intervention	Medication adherence	20 RCTs	HCPs	Educational interventions led by HCPs (13 RCTs)	Not conducted due to high heterogeneity	Moderate
van Driel et al. (2016)	CVD	Effects of interventions aimed at improving adherence to lipid‐lowering medications	Usual care	Medication adherence Clinical outcomes	35 RCTs	HCPs	7 studies compared adherence rates of those in an intensification of a patient care intervention (e.g., electronic reminders, pharmacist‐led interventions, healthcare professional education of patients) versus usual care	7 studies Participants in the intervention group had better adherence than those receiving usual care (odds ratio = 1.93, 95% CI 1.29 to 2.88	Moderate
Ryan et al. (2014)	Various	Interventions to improve safe and effective medicines use	Unrestricted	Medication use, medication adherence, adverse events and clinical outcomes	75 systematic reviews	Pharmacists	“Simplified dosing regimens: with positive effects on adherence” “Interventions involving pharmacists in medicines management, such as medicines reviews (with positive effects on adherence and use, medicines problems and clinical outcomes) and pharmaceutical care services (consultation between pharmacist and patient to resolve medicines problems, develop a care plan and provide follow‐up; with positive effects on adherence and knowledge)” “Education/information as part of pharmacist‐delivered packages of care”	Not conducted due to high heterogeneity	Moderate
Brown et al. (2019)	Depression	Pharmacy-led interventions to support patients with depression	Usual care	Depression Medication adherence	12 studies	Pharmacists	6 studies did show that people who received support from their pharmacy were more likely to take their antidepressants as prescribed	Risk ratio = 0.73, 95% CI 0.61–0.87)	High
Mateo‐Urdiales et al. (2019)	HIV	effects of interventions for rapid initiation of antiretroviral therapy (defined as offering antiretroviral therapy within 7 days of HIV diagnosis)	Usual care	Medication uptake Clinical outcomes	7 studies	HCPs	“The rapid antiretroviral therapy intervention was offered as part of a package that included several cointerventions targeting individuals, health workers and health system processes delivered alongside rapid antiretroviral therapy that aimed to facilitate uptake and adherence to antiretroviral therapy”	4 studies Better antiretroviral therapy uptake at 12 months (risk ratio = 1.09, 95% CI 1.06 to 1.12	Moderate
Weeks et al. (2016)	Various	To assess clinical, patient‐reported, and resource use outcomes of non‐medical (nurses, pharmacists, allied health professionals, and physician assistants) prescribing for managing acute and chronic health conditions in primary and secondary care settings compared with medical prescribing (usual care)	Medical prescribing	Medication adherence Clinical outcomes	46 studies	HCPs	4 studies - continuous outcome data showed an effect favoring patient adherence in the non‐medical prescribing group	(Mean difference = 0.15, 95% CI 0.00–0.30)	Moderate
Smith et al. (2021)	Various	‐oriented interventions designed to improve outcomes in people with multimorbidity in primary care and community settings	Usual Care	Medication adherence Clinical outcomes Healthcare utilization HCP behaviour	17 RCTs	HCPs	“In 11 studies, the predominant intervention element was a change to the organization of care delivery, usually through case management or enhanced multidisciplinary team work. In six studies, the interventions were predominantly patient‐oriented, for example, educational or self‐management support‐type interventions delivered directly to participants”	4 studies, slightly improve medication adherence	Low
Gordon et al. (2023)	Inflammatory bowel disease	Different types of educational interventions, how they are delivered, and to determine their effectiveness and safety in people with inflammatory bowel disease	Usual care	Medication adherence Clinical outcomes	14 studies	HCPs	HCP-led training programs	Medication adherence, patient knowledge and change in quality of life showed conflicting results that varied between no major differences and differences in favor of the educational interventions	Low

Notes. CVD, cardiovascular disease; GRADE, grading of recommendations assessment, Development and Evaluation; HCP, healthcare provider; RCT, randomized controlled trial.

Across systematic review studies, HCP-delivered medication adherence interventions typically produce small-to-medium effect sizes for adherence outcomes (e.g., pharmacy-led interventions to support medication adherence in diabetes, standardized mean difference effect size = −0.68; 95% CI -0.79, −0.58; *p* < 0.001 (Presley et al., 2019); HCP-led interventions to support medication adherence in acute coronary syndrome, odds ratio = 1.54, 95% CI 1.26, 1.88, *p* < 0.001 (Crawshaw et al., 2017)), however, there is considerable heterogeneity in terms of sample population, intervention type, and study outcomes. Moreover, few interventions that show promise in improving adherence are powered to test their effect on clinical outcomes (Nieuwlaat et al., 2014) or are implemented into routine practice which contributes to evidence-to-practice gaps. Wilhelmsen and Eriksen conducted a systematic review of reviews around this topic which included 32 systematic reviews of varying methodological quality (Wilhelmsen and Eriksson, 2019). A total of eight systematic reviews, five of which were Cochrane systematic reviews, were rated as high-quality and were further analyzed. Some key findings from their analysis revealed that patient education and counselling (e.g., information to help patients understand what the medication is doing in the body, are addressing patient concerns that commonly occur such as worries about side effects or long-term impacts of taking a medication) showed some positive effects on medication adherence. Simplifying medication dosing was shown to have some benefit on morbidity and patient satisfaction. Interventions delivered by pharmacists and nurses were more effective than interventions delivered by primary care physicians. Similar findings were reported by Ryan and colleagues who conducted a Cochrane systematic review of 75 reviews evaluating the effects of interventions to improve medication adherence. In relation to features of HCP-delivered interventions, there was evidence that simplifying medication dosing and interventions involving pharmacists had generally positive effects on medication adherence (Ryan et al., 2014).

In the next section, we posit some key features of potentially effective HCP-delivered interventions to support medication adherence.

### 3.1 Moving beyond education-only

Patient education is a commonly used strategy to support adherence and HCPs are in a good position to deliver these types of interventions due to their established trusting relationship and ongoing contact with patients. However, whilst education is necessary for behaviour change (the individual needs to know about what they are meant to do and why it is important to do it), it may not be sufficient on its own to support meaningful behaviour change over time. Education can certainly help support a patient make sense of their medication regimen by addressing beliefs about their illness and/or treatment (perceptual barrier) yet other considerations may be required if a patient is experiencing practical barriers to adherence. A systematic review of reviews by Anderson and colleagues identified several adherence intervention components beyond education (focusing on practical barriers to medication-taking) that include simplifying medication dosing (e.g., reducing the number of medications or instances per day which medications are taken), electronic and non-electronic reminders, incentives to reduce out-of-pocket costs, monitoring and feedback, habit-focused interventions, and specialized medication packaging. Notably, interventions were found to be more effective when they included multiple strategies (Anderson et al., 2020). Whilst education may often be seen as the ‘default’ strategy (it is clearly important), it is crucial that HCPs have a variety of tools in their professional ‘toolbox’ to meet the needs of their patients.

### 3.2 Using multiple behaviour change strategies

It is expected that HCPs should have multiple behaviour change strategies at their disposal to support patients to be adherent over time (see medication adherence clinical practice guideline from the UK’s National Institute for Health and Care Excellence (NICE) (National Institute for Health and Care Excellence, 2009)). In line with the PAPA outlined above, patient education and counselling (e.g., telling a patient about what medication they will be prescribed and answering any questions or concerns they might have) may be a helpful strategy for patients that report ambivalence towards their medications or have concerns about potential side effects (i.e., perceptual barrier, intentional non-adherence (Horne et al., 2019)). However, other strategies may be required for individuals who are motivated but experience other barriers to adherence, such as forgetting to take treatment regularly or having complex drug regimens to manage (i.e., practical barrier, unintentional non-adherence (Horne et al., 2019)). It should, however, be noted that intentional barriers (e.g., medication beliefs) and unintentional barriers (e.g., forgetting) may not be mutually exclusive, with some evidence that intentional non-adherence mediates unintentional non-adherence (Gadkari and McHorney, 2012).

### 3.3 Tailoring interventions to different determinants of non-adherence

In addition to the need for multiple behaviour change strategies to be available for HCPs, it is also probable that tailoring the strategy to the patient and the issues they are facing is required for the best possible results. As highlighted by Bosworth and colleagues (Bosworth et al., 2011), given the myriad factors associated with medication adherence, it is unreasonable to think that a one-size-fits-all approach would be appropriate. It should be acknowledged that adding in aspects of tailoring to HCP-delivered adherence intervention undoubtedly increases complexity of such interventions, however, this is likely the price to pay in order to maximize effectiveness. Allemann and colleagues suggested that medication adherence interventions should target current modifiable factors and be tailored to unmodifiable factors (Allemann et al., 2016). For example, a HCP-delivered intervention targeting medication beliefs posing barriers to adherence (a potentially modifiable factor) tailored to the individuals level of education and ethno-cultural background (an unmodifiable factor), may be a more suitable approach versus a standardized, non-tailored approach.

### 3.4 Involving pharmacists and nurses as intervention deliverers

Multiple HCP groups such as physicians, nurses, and pharmacists could conceivably be integrated into delivering adherence interventions, which is reflected in the literature (Nieuwlaat et al., 2014; Crawshaw et al., 2019) There is evidence to suggest that some HCPs may be better placed than others to deliver adherence interventions. Two systematic reviews of reviews by Wilhelmsen and Eriksen (Wilhelmsen and Eriksson, 2019) and Ryan and colleagues (Ryan et al., 2014) found that interventions delivered by pharmacists and nurses showed a better result in improving adherence and outcomes than interventions delivered by primary care physicians. Reasons may include more frequent and sustained patient contact among allied HCPs versus physicians, greater involvement in follow-up care, and specific training in techniques such as motivational interviewing (Nieuwlaat et al., 2014). Pharmacists have seen a shift in practice in recent years in an attempt to increase patient-facing activities (e.g., spending more time talking to patients about their medication regimen). This enhanced role of pharmacists to interact with patients directly patient directly using education and counselling methods, provides an opportunity for better supports to be in place for patients over time (Kini and Ho, 2018). Importantly, the effectiveness of HCP-delivered interventions may also vary by care setting. Community pharmacists, for example, often have more frequent and informal contact with patients, which facilitates timely adherence discussions and follow-up. In contrast, hospital-based teams may benefit from access to multidisciplinary support and clinical data, but have fewer opportunities for sustained patient engagement post-discharge. These contextual differences should inform how adherence interventions are designed and which HCPs are best positioned to deliver them. Successful integration of pharmacists, nurses, and physicians into multidisciplinary adherence teams depends on factors such as clearly defined roles, effective communication workflows, and shared accountability. According to the Interprofessional Collaboration Model (Orchard et al., 2010), high-functioning teams require mutual respect, common goals, and structured coordination mechanisms. However, practical barriers can undermine collaboration, including hierarchical dynamics and reimbursement models that may undervalue the contributions of certain HCP groups. As discussed in Section 5, addressing these system-level challenges is critical to enabling scalable, team-based adherence support.

### 3.5 Providing ongoing support to patients across stages of adherence

Medication-taking for chronic conditions is often long-term/lifelong behaviour. As such, it is likely that HCPs need to be available to provide ongoing support for patients at various timepoints which requires synchrony across acute, primary, and community care settings. It may be that different HCPs are more involved at different times (and at multiple timepoints) during the patient’s journey, when acute hospital events transition into primary and community care. For example, HCPs working in primary care settings are well-placed to identify patients who do not initiate treatment. Care mechanisms should be in place to support patients as they transition between services (Tyler et al., 2023; Daliri et al., 2021). This is particularly pertinent in the post-discharge period from hospital when issues around medications (e.g., side effects) can arise and can lead to premature discontinuation. Odeh and colleagues address this issue nicely as part of a pharmacist-led, post-discharge intervention study to support medication use among polypharmacy patients (Odeh et al., 2019). The intervention comprised multiple telephone touchpoints between pharmacist and patient within three-months of hospital discharge with tailored conversations informed by the PAPA. The study found that patients receiving the intervention had better adherence and lower readmission rates versus those in a propensity score matched control group. Moreover, the intervention was associated with greater cost-effectiveness. This study demonstrates several features discussed so far, namely, tailoring intervention content using a theory such as the PAPA, using pharmacists to deliver interventions, and providing post-discharge at multiple timepoints.

### 3.6 Addressing health system-level barriers and inequities

Many of the points detailed above speak directly to the practice of HCPs. However, HCPs operate as part of a health system where other macro-level challenges sometimes make it difficult for HCPs to adequately support patients with their medication-taking. Health system barriers such as access to services, available resources, time, and cost associated with clinical practice can all potentially impact how HCPs support patients with their treatment which can also exacerbate health inequities among patients. Moreover, given the multitude of factors relating to adherence (e.g., patient-, disease-, therapy-, socioeconomic-, and healthcare system-related factors (Sabaté, 2003)), it seems likely that multifaceted interventions are most appropriate, despite the inherent difficulty of implementing complex interventions into routine practice. Much of this multi-layered and multi-component intervention thinking speaks to the use of models, theories, and frameworks from the literature to better inform the development, evaluation, and implementation of complex adherence interventions (Conn et al., 2016a) (a topic discussed further in Section 6).

## 4 Implementation approaches targeting evidence-to-practice gaps among HCPs supporting medication adherence

We conceptualize a HCP-targeted adherence intervention as one that is focusing on HCP clinical practice (i.e., implementation intervention), to essentially help HCPs to help their patients to be more adherent to treatment. The key feature here is the primary focus on HCP behaviour rather than a patient, given that HCPs are considered the recipient of the intervention itself. Identifying such gaps in clinical practice and focusing on behaviour change among HCPs speaks directly to the field of implementation science (Grimshaw et al., 2012). As such, we can use learnings and evidence from implementation science to help understand why evidence-to-practice gaps occur and how such gaps can be addressed in real world settings. To date, there have been several systematic reviews conducted in this area focusing on HCP practice change interventions and implementation strategies to support knowledge uptake (e.g., Clinical practice guidelines).

A systematic review of 218 HCP-targeted intervention studies found small improvements in patient adherence (mean difference effect size = 0.23; 95% CI 0.19, 0.29; *p* < 0.001) (Conn et al., 2015). Specific types of HCP-targeted interventions included improving HCP medication adherence skills (e.g., teaching HCPs how to uncover patients’ barriers to adherence and generate solutions), integrating healthcare processes (e.g., strategies designed to improve care coordination between HCPs), improving HCP communication skills, providing feedback to HCPs about patients’ adherence, HCPs monitoring adherence, shared decision-making, increasing time with patients, and reducing distance between patients and their HCP (mean difference effect sizes ranged from 0.01–0.30 between types of interventions). Subgroup analyses did not find certain types of interventions to be superior than others, however, mediation analysis revealed that interventions were more effective when they included multiple strategies. A limitation of these data were that most intervention studies did not measure or report actual changes to HCP clinical practice which limits our understanding of how these types of interventions work (i.e., in conceptualizing pathways to change, it would be expected that such interventions change HCP behaviour which then leads to patient behaviour change (Toomey et al., 2020)).

In the next section, we posit some key features of potentially effective HCP-targeted adherence interventions and offer some system-level implementation approaches for addressing known evidence-to-practice gaps (see Table 2 for a summary of key features of HCP-delivered versus HCP-targeted adherence interventions).

**TABLE 2:** Key features of potentially effective HCP-delivered interventions (Section 4) and HCP-targeted implementation approaches (Section 5) to support medication adherence.

Feature	HCP-delivered interventions (Section 4)	HCP-targeted implementation approaches (Section 5)
Primary Aim	Improve patient medication adherence	Improve HCP practice related to adherence support
Intervention recipients	Patients	HCPs
Intervention delivery agents	Physicians, pharmacists, nurses	Health systems, implementation teams
Intervention examples	Education, counselling, simplified dosing, reminders, pharmacist-led post-discharge support	Audit and feedback, educational meetings, clinical reminders, local champions
Design features	Use of multiple behavior change strategies; tailoring to perceptual/practical barriers (PAPA); ongoing patient contact	Based on implementation science strategies; often includes multifaceted approaches requiring system changes
Key barriers	Patient-level barriers (e.g., ambivalence, forgetfulness, complex regimens)	HCP-level barriers (e.g., lack of knowledge, workflow challenges, lack of feedback)
Key facilitators	Trust, personalization, repeated contact	Role clarity, actionable guidelines, interprofessional collaboration, technology support
System-level supports	Coordination across care settings; inclusion of pharmacists and nurses	Integration into workflows; electronic medical record alerts; adherence screening tools; incentive and remuneration models

### 4.1 Early identification and routine screening of adherence issues

A systemic issue within health systems is the lack of streamlined processes to recognize adherence issues early (e.g., patients not initiating treatment) and to routinely screen for poor adherence. If HCPs are unaware of adherence issues, then it remains difficult to initiate supports for patients to reduce the likelihood of treatment discontinuation. In the simplest terms, screening might involve HCPs asking patients about their medication-taking behaviour in an honest and open way (as indicated in the UK’s NICE guidelines for medication adherence (National Institute for Health and Care Excellence, 2009)), normalizing challenges with adherence (e.g., “many people find it difficult to take meds regularly … ”), and referring to specific time periods when discussing medication use (e.g., ‘over the past month …). Specific issues relating to early identification/screening include a lack of valid screening tools, inadequate integration of existing tools into electronic medical record systems, as well as time pressure and a lack of expertise, all of which reduce the likelihood that adherence issues are screened for and then discussed in an open and honest way (Garfield et al., 2011; Engel et al., 2017). Medication adherence screening tools along with more general patient-reported outcome/experience measures should be embedded into routine practice and HCPs should be trained on their use and provided opportunities to practice using them (Stirratt et al., 2015; Gleeson et al., 2016). Advances in health technology may offer promising solutions to some of these issues: for instance, artificial intelligence (AI)-driven risk prediction algorithms can flag patients likely to experience adherence issues using electronic medical records or pharmacy data (Babel et al., 2021). Digital tools such as mobile applications with HCP dashboards (e.g., Medisafe) also provide real-time monitoring capabilities, allowing HCPs to track missed doses and initiate timely support (Babel et al., 2021; Hartch et al., 2024) These digital tools may also help address systemic barriers by automating parts of the adherence screening process, reducing the time-burden on HCPs, and potentially improving scalability of routine adherence monitoring across large patient populations.

### 4.2 Making adherence-related clinical practice guidelines more actionable

Clinical practice guidelines are crucial to identify evidence-to-practice gaps to inform the clinical practice of HCPs. Ruppar and colleagues conducted a systematic review of 23 clinical practice guidelines to identify recommendations relating to medication adherence (Ruppar et al., 2015). Key recommendation categories included assessment strategies, educational strategies, behavioural strategies, therapeutic relationship strategies, and outside influences/co-morbidities. The authors called for additional rigor for developing these types of guidelines and also suggested that the strategies listed in the guidelines were too vague and lacked specific, workable examples to guide HCPs; thus, making recommendations in the guidelines hard to operationalize in practice. Moreover, dissemination plans across the guidelines were often suboptimal or missing entirely, meaning that engagement with target HCPs may be impacted.

Clinical practice guidelines are only useful if they are adopted by those they are targeting. Thus, it may be useful to embed a behaviour change perspective into the guideline development process, or into the development of an implementation intervention that is intended to support the integration of an existing guideline into practice. In a critical appraisal of guideline recommendations which identified behavioural specification as the foundational element for implementation, none of the included recommendations were fully behaviourally specific, and there was a lack of consistency on required behaviours across guidelines for the same topic (Graham et al., 2023). Multiple systematic reviews identify lack of specificity of guideline recommendations as a key barrier to their uptake (Wang et al., 2023). Additional work could be done to specify individual guideline recommendations in behavioural terms (i.e., clarify the specific clinical action to be undertaken along with who should do it, when, where, and how (Michie and Johnston, 2004)). This could be achieved using the Action, Actor, Context, Target, Time (AACTT) Framework, developed to support behavioural specification in implementation studies (Presseau et al., 2019), but which could be applied to help improve how clinical practice guideline recommendations are written (Michie and Johnston, 2004). The framework defines five components that should be specified to fully describe a behaviour that is being targeted for change in healthcare contexts, namely,: the “Action” (a discrete observable behaviour); “Actor” (the individual or group of individuals who perform (or should/could perform) the action; ‘Context’ (the physical setting in which the actor performs (or should/could perform) the action; “Target” (the individual or group of individuals for/with whom the actor performs the action; and “Time” (the time period and duration that the actor performs the action in the context with/for the target) (Presseau et al., 2019). In addition, clinical practice typically doesn’t change based on guideline dissemination alone; active implementation strategies are typically required to encourage the desired change (Grimshaw et al., 2012; Crawshaw et al., 2025).

### 4.3 Using routinely collected data to identify patients with adherence issues

The widespread and persistent issue of poor medication adherence lends itself to large scale, population-based research methods and the harnessing of ‘big data’. Patients reliant on medications are tied to a range of care settings and stakeholders including the prescriber’s clinic, the dispensing pharmacy, their health plan, prescription drug plan, and pharmacy benefit management, which requires system-level synergy to reduce gaps in care (Bosworth et al., 2016). From the perspective of a HCP, having up-to-date medication-related information and data linkages between prescribing and dispensing services may help to identify patients at risk of non-adherence. Again, this provides an example of the context and systems infrastructure in which HCPs work which can enhance or inhibit their ability to address medication adherence issues among their patients.

### 4.4 Enhancing HCP incentivization models to support adherence

Incentivization for providing services (e.g., pay-for-performance) is commonplace in health systems, however, HCP activities related specifically to medication adherence are not routinely incentivized, and for those that are, may be unbalanced to favor certain HCP groups over others. Established prescribing services such as the “New Medicines Service” and “Medicines Use Review” programs in the United Kingdom have shown to add clinical value in primary care and community pharmacy contexts (Elliott et al., 2020), however, activities targeting medication adherence specifically are yet to be established across the board (Khan and Socha-Dietrich, 2018). As such, there have been calls to expand HCP remuneration models to capture activities focused on identifying and addressing adherence issues and capturing adherence data over time, which may encourage practice change and improved medication adherence management.

### 4.5 Establishing quality indicators for adherence monitoring and support

In addition to incentivizing adherence-related activities among HCPs, there may also be an argument to develop care quality indicators around medication adherence (i.e., adherence as a performance measure). In practice, this would involve setting evidence-based benchmarks around the delivery of adherence-related services in routine practice (e.g., screening rates for non-adherence, community pharmacy referrals to discuss adherence issues). This could potentially set the stage to leverage knowledge from the audit and feedback literature to support medication adherence-related clinical targets and improve processes of care (Zaugg et al., 2018).

### 4.6 Drawing on what is already known about supporting practice change from implementation science

There are opportunities to draw on the broader implementation science literature to inform the design and evaluation of HCP behaviour change-focused interventions to better support patient medication adherence. For example, numerous systematic reviews have been produced which have established the effectiveness of specific implementation strategies such as educational meetings, audit and feedback, clinical reminders, and local champions who drive change (Ivers et al., 2012; Pantoja et al., 2019; Forsetlund et al., 2021). Systematic reviews tend to show that such implementation interventions lead to small-to-medium improvements in clinical practice, and these can be a good place to start when considering which implementation strategy to pursue. The variation in effectiveness often identified indicates that more work needs to be done to determine how to maximize the effectiveness of such interventions. Such work is ongoing across the field and is relevant to the development of adherence-focused interventions. For example, evidence indicates that audit and feedback is more likely to be effective when the feedback is provided more than once, when it is relayed by a supervisor or colleagues, is delivered in a written format accompanied by verbal feedback, and when it includes both explicit targets for change and an action plan for achieving those targets (Ivers et al., 2012, 2025). A recent systematic review focusing on the pharmacist role in primary care found that involving pharmacists in the delivery of audit and feedback interventions can lead to improvements in prescribing outcomes, providing both verbal and written feedback enhances effectiveness, and also determined that the addition of computerized decision support for prescribers led to greater practice improvements (Carter et al., 2023).

Similar to patient behaviours, the determinants of HCP behaviours are wide-ranging and their relative importance as targets for change may vary depending on several factors including the nature of the behaviour under focus and the wider context in which it is enacted. Previous research has identified several important factors which can influence HCP behaviour, including knowledge of guideline recommendations (Beenstock et al., 2012); social influences, professional roles and identities, and power dynamics (Etherington et al., 2021); having multiple goals for care delivery which may facilitate or conflict with one another (Presseau et al., 2009); the strength of intention to perform specific clinical behaviours (Godin et al., 2008); and the extent to which clinical behaviours are habitual or can be performed relatively automatically (Presseau et al., 2014; Potthoff et al., 2019). Many clinical behaviours become highly routinized over time, and there have been several calls in the literature for more studies that incorporate dual process models and seek to understand the role of automatic determinants of HCP behaviour alongside reflective determinants (Nilsen et al., 2012). To develop appropriately-targeted interventions for HCPs, further work is needed to understand which HCP behaviours are key for supporting medication adherence and the factors that influence these behaviours in the various contexts in which HCPs work.

Drawing on existing evidence such as this when developing interventions can help to maximize the impact of HCP-focused strategies to improve medication adherence. A key tenet of implementation science is the importance of developing a detailed understanding of the problem before selecting and implementing an intended solution. This can help to ensure that the selected strategy is fit-for-purpose and adequately addresses existing barriers to or facilitators of change. For instance, time constraints (opportunity-related issue), guideline familiarity (knowledge issue), and habitual prescribing patterns (automaticity issue) are known barriers to practice change, yet each would require markedly different strategies to support HCPs to change their behaviour. Frameworks such as the Theoretical Domains Framework (Cane et al., 2012) or the Consolidated Framework for Implementation Research (Damschroder et al., 2022) can be used to systematically identify the barriers and facilitators for a specific medication adherence-related practice issue to form the basis for intervention development and increase the chances of success.

## 5 Future directions and recommendations for research and practice

Given the complexity of medication adherence as a behaviour, it is perhaps unsurprising that there is considerable heterogeneity across HCP adherence interventions in terms of sample population, intervention type/content/delivery, and study outcomes, thus making it difficult for adherence researchers to navigate through the evidence landscape. In recent times, there has been progress to improve the reporting of the content, delivery and other features of behaviour change interventions using theory-informed tools such as the Behaviour Change Technique Taxonomy version 1 (BCTTv1) (Michie et al., 2013) and more recently the Behaviour Change Intervention Ontology (BCIO) (Norris et al., 2019) to aid intervention development, evaluation, and optimization. Another useful approach is Intervention Mapping (IM) (Kok et al., 2016). This approach involves: conducting a needs assessment to identify target behaviours and behavioural determinants (Sabaté, 2003); identify determinants to target for change by mapping behaviours to their determinants to create matrices of change objectives (Kardas et al., 2024); select and operationalize theory-based intervention components to address identified determinants (Kardas et al., 2013); develop an organized program based on the intervention components (MacIntyre et al., 2005); plans for adoption, implementation, and sustainability (Miller et al., 2002); develop a plan for outcome and process evaluation. The IM approach provides a taxonomy of behaviour change techniques and a process within which theory can be integrated. It has been used to develop adherence interventions targeting both intentional and unintentional non-adherence (Moon et al., 2021). One of the overarching goals of using frameworks such as the BCTTv1, BCIO, or an IM approach is to ensure a higher success rate of behaviour change interventions. Can we get the point where we can empirically state ‘which behaviour change techniques work for whom in which contexts delivered by what means’ (Armitage et al., 2021)? This line of questioning closely relates to 3Cs reported by Horne and colleagues (Content–what is being delivered?; Channel–how is it being delivered?; and Context–what is the setting/circumstance in which delivery happens (Horne et al., 2019; Stewart et al., 2023)). In terms of medication adherence research, this line of work has the potential to help develop HCP interventions that are more behaviourally intelligent because their content is based on sound understanding of adherence and based on evidence rather than rolling out the same ideas which have been shown to be generally ineffective.

There is encouraging work progressing in relation to medication adherence study outcomes with the recent development of a core outcome set for medication adherence trials in primary care (Bhattacharya et al., 2024). Whilst green shoots of progress are most welcomed in this space, there remains a persistent challenge, namely, that few interventions that show promise are implemented, scaled, and costed within health systems. Therefore, additional emphasis must focus on the cost-effectiveness of effective HCP adherence interventions and their scalability. Moreover, we reiterate the need for medication adherence research to consider the dual roles of HCPs as both deliverer and target of behaviour change interventions. Work should be done to understand the barriers to behaviour change among HCPs and what can then be done to support implementation. This should involve working closely with HCPs to understand their perspectives about what factors might impede implementation efforts and generating ways around such barriers. We have highlighted a number of areas where HCP practice can directly support patients to adhere to treatment (e.g., moving beyond education-only strategies to a ‘toolbox’ of distinct, tailorable strategies), however, it is imperative that HCPs are supported at a system-level to allow them to improve their practice. Developing and integrating adherence screening tools, improving clinical practice guidelines, adapting health technology infrastructure, and generating quality indicators are just some examples of system-level solutions which are probably needed to shift the needle to improve both medication adherence and clinical outcomes and support implementation efforts in the real world.

## 6 Conclusion

There is an extensive evidence-base for the effectiveness of HCP-delivered interventions to support medication adherence, and a growing evidence-base for approaches targeting practice change among HCPs. We have identified several areas that could help advance both research and clinical practice with a particular focus on the content and delivery of HCP adherence interventions, the implementation of effective strategies, and the need for system-level approaches to support HCPs. We believe there is opportunity to leverage learnings and evidence from implementation science to help support the uptake and scale of effective adherence interventions into routine practice.
