# Correction: Healthcare provider interventions to support medication adherence: state-of-the-science overview

**DOI:** 10.3389/fphar.2026.1826521

**Published:** 2026-03-16

**Authors:** Jacob Crawshaw, Nicola McCleary

**Affiliations:** 1 Methodological and Implementation Research Program, Ottawa Hospital Research Institute, Ottawa, ON, Canada; 2 Child Health Evaluative Sciences Program, The Hospital for Sick Children, Toronto, ON, Canada; 3 Institute of Health Policy, Management and Evaluation, University of Toronto, Toronto, ON, Canada

**Keywords:** medication adherence, behaviour change, healthcare providers, implementation science, health systems, state-of-the-science

The funder Abbot Pharmaceutical Ltd. was erroneously omitted. The correct Funding statement reads:

The author(s) declared that financial support was received for this work and/or its publication. The journal publication fee for this article was paid by Abbot Pharmaceutical Ltd. The funder was not involved in the study design, collection, analysis, interpretation of data, the writing of this article, or the decision to submit it for publication.

The original article has been updated.

